# Physics-Driven Deep Feature Fusion: A Lightweight CSAKansformer Architecture for Tool Wear Diagnosis in P25 Turning

**DOI:** 10.3390/s26102937

**Published:** 2026-05-07

**Authors:** Shuqiang Wang, Tianyue Zhang, Ximin Liu, Wei Liu, Huanqi Zhang, Feng Chang

**Affiliations:** School of Mechanical and Power Engineering, Shenyang University of Chemical Technology, Shenyang 110142, China

**Keywords:** multi-source fusion, deep learning, tool wear identification

## Abstract

Accurate tool wear identification is essential for ensuring the continuity of intelligent machining and workpiece quality. To address the challenges of multi-source fusion inefficiency and inadequate feature extraction, this study proposes a novel identification architecture combining physics-guided multi-channel Gramian angular field (PG-MGAF) with a minimalist 14-layer CSA-Kansformer network. Multi-source signals are preprocessed via PG-MGAF to convert 1D time-series into 2D RGB images, effectively characterizing spatial coupling and interactive energy across three channels. Subsequently, the minimalist network maps these composite features to tool states, significantly reducing computational overhead. Experimental results demonstrate that the proposed model achieves an average accuracy of 93.6% with a single-step inference latency of only 5.90 ms, significantly outperforming mainstream methods such as MobileNet-V2 and ConvNeXt. This architecture provides a high-efficiency, low-latency solution for real-time tool condition monitoring under complex industrial conditions.

## 1. Introduction

With the deep advancement of Industry 4.0 and intelligent manufacturing, high-speed, high-precision, and unmanned machining impose higher requirements on the modern equipment manufacturing industry. The inevitable wear or sudden failure of cutting tools during continuous operations directly leads to workpiece rejection, shortened equipment lifespan, and severe unplanned production interruptions. Statistics show that tool-related costs account for 25% to 30% of total machining costs, and downtime caused by tool failures constitutes 1/3 of total automated line downtime [1]. Therefore, achieving accurate, real-time tool condition monitoring (TCM) is essential. Existing TCM technologies are divided into offline inspection and online monitoring. Offline inspection is inefficient and difficult to adapt to continuous automated production due to required machine shutdowns [2]. While online monitoring is the mainstream, direct visual measurement is highly susceptible to harsh working conditions like cutting fluid splashing and chip occlusion. In contrast, indirect monitoring methods based on physical signals—such as cutting force, vibration, and acoustic emission—effectively overcome these limitations. Offering real-time, non-destructive, and uninterrupted tracking, they have become the most widely applied technical route in TCM [3].

Current indirect monitoring-based TCM research mainly branches into two paradigms. The first is end-to-end deep learning based on one-dimensional (1D) time-series signals. Employing architectures like 1D-CNN, Temporal Convolutional Networks (TCN), and Long Short-Term Memory (LSTM) networks, this branch directly extracts features from raw signals, eliminating reliance on manual feature engineering [4]. However, the restricted local receptive field of 1D convolutions struggles to simultaneously capture global temporal dependencies and local high-frequency mutations. Consequently, weak early-stage wear signals are easily submerged by intense operational noise [5,6]. Furthermore, the fusion of multi-source heterogeneous signals is often limited to shallow channel concatenation, failing to uncover coupled evolution mechanisms among different physical signals [7,8]. The second branch is the visual recognition method based on the two-dimensional (2D) imaging of 1D signals. Through specific encoding rules, 1D signals are converted into 2D images, transforming hidden wear characteristics into visual features such as texture, grayscale, and spatial structure for end-to-end recognition via mature computer vision models [9]. This fully exploits the advantages of multi-scale feature extraction and global receptive fields, simultaneously capturing global evolution patterns and local mutation features [10]. It provides a high-dimensional representation space for deep multi-source fusion, offering a novel pathway to break through the inherent limitations of the 1D route [11].

Recent studies have extensively explored 2D imaging and feature extraction for single-sensor time-series signals, with technological evolution predominantly centering on imaging method optimization and network architecture upgrades. For instance, Martínez-Arellano et al. pioneered the use of the Gramian angular summation field (GASF) to losslessly encode 1D cutting force signals for CNN-based classification, laying the foundation for 1D-to-2D signal conversion. To capture high-frequency transients indicative of early wear, the Continuous Wavelet Transform (CWT) has been widely adopted [12]. Subsequent studies integrated CWT with multi-scale feature pyramids [13], improved ResNets [14], and MobileViT [15] to balance recognition accuracy and low-latency online monitoring. Despite these advances, single-sensor imaging exhibits inherent limitations. A solitary imaging mechanism captures only one facet of the signal (e.g., GASF emphasizes temporal dependencies, while CWT focuses on time-frequency distributions), failing to comprehensively depict the non-stationary wear evolution. Furthermore, single-sensor data cannot span the full degradation spectrum—from macroscopic load shifts to microscopic material fractures—making it highly vulnerable to working condition fluctuations and industrial noise.

To overcome single-sensor representation bottlenecks, multi-source fusion imaging has emerged as a crucial research frontier. Researchers have attempted to expand feature dimensions by combining multiple imaging mechanisms and sensor modalities. For example, methods integrating GASF and Markov transition fields (MTF) [16], time-frequency MTF [17], and deep domain adaptation networks [18] have demonstrated improved accuracy and robustness under variable conditions. Kou et al. further pushed this boundary by concatenating GAF-encoded time-series features with infrared thermal images [19]. Nevertheless, existing multi-source fusion strategies have yet to resolve fundamental pain points. First, current fusion paradigms predominantly rely on horizontal concatenation or shallow channel stacking. By ignoring the inherent physical topological correlations and coupled evolution mechanisms of heterogeneous signals (e.g., force, vibration, and acoustic emission), these methods fail to achieve deep, channel-level energy interactions. This often amplifies wear-irrelevant noise and degrades overall robustness. Second, the absence of a unified, full-life-cycle health baseline normalization framework makes it difficult to isolate wear-specific degradation increments from amplitude disparities across varying sensors, thereby leaving early weak wear features susceptible to noise submergence.

Regarding feature extraction networks, while the introduction of CNNs, Transformers, and attention mechanisms has elevated performance, two critical bottlenecks persist. First, feature mapping in mainstream architectures heavily relies on multi-layer perceptrons (MLPs). Given that tool wear is a strongly coupled, highly non-linear dynamic process, the static “linear weights + fixed activation” mechanism of MLPs struggles to accurately approximate complex degradation boundaries. Second, conventional attention mechanisms are tailored for general visual images and are fundamentally misaligned with physically encoded multi-channel fused images. They fail to adaptively focus on wear-critical feature flows, resulting in the severe underutilization of physical degradation information.

To address the bottlenecks of multi-source feature fusion and non-linear feature extraction under complex working conditions, and to achieve high-accuracy identification of tool wear states, the main contributions of this paper are as follows:

1. A WGAN-DIV-based minority-class augmentation and high-dimensional physical map reconstruction mechanism oriented toward complex turning conditions is proposed. Addressing the issues of sample imbalance across wear stages and the limited representation capability of single-channel imaging, this paper first introduces a divergence-regularized Wasserstein generative adversarial network (WGAN-DIV) to generate minority-class samples for the slight-wear and severe-wear stages. The generated samples are further evaluated through MMD, SSIM, and statistical consistency tests to verify their distributional fidelity. Subsequently, the augmented multi-source features are physically rearranged and adaptively normalized according to the “force–vibration–acoustic emission” hierarchy, and then mapped into three RGB channels, namely R: static spatial coupling based on GASF, G: dynamic degradation divergence based on GADF, and B: energy compensation based on cross OPM. This mechanism enhances the physical coherence of the input representation and improves the robustness of subsequent wear-state recognition.
2. A minimalist recognition model (CSA-Kansformer) integrating physical prior attention and the KAN architecture is constructed. Aiming at the extremely non-linear degradation process of tool wear, this paper introduces the cutting-edge KAN architecture to replace the traditional fixed MLP module, relying on its powerful spline fitting advantages to accurately approximate high-order non-linear features. Simultaneously, the model embeds a channel-spatial attention mechanism (CSAM), which can adaptively evaluate the contribution of each heterogeneous physical channel and dynamically allocate weights based on the pre-established RGB physical encoding. This architecture effectively broadens the non-linear decision boundary for extracting extreme wear features and substantially reduces computational overhead while ensuring a minimalist network depth.
3. Generalization validation and engineering implementation evaluation based on a real turning full-life-cycle dataset are conducted. Breaking through the limitations of existing research that mostly relies on a single, ideal milling dataset, this paper introduces an industrial turning dataset that deeply integrates machine tool control system data, external multi-source sensor data, and high-precision actual wear labels for validation. Ultimately, a novel identification framework integrating high confidence and strong robustness is established. It not only effectively overcomes the non-linear classification challenges under complex working conditions but also provides a solid data-driven foundation for the precise early warning and practical implementation of extreme tool wear in intelligent machining production lines.

## 2. Theoretical Bases

### 2.1. GAF

The Gramian angular field (GAF) is an encoding method that maps one-dimensional time-series signals into two-dimensional feature images [20]. This method can fully preserve the temporal dependencies and topological structure of the original signals within a high-dimensional space, serving as an effective means to achieve the visualization of time-series signals. The core construction process of GAF primarily comprises three steps: signal normalization, polar coordinate mapping, and Gram matrix computation.

First, to satisfy the domain requirements of the subsequent trigonometric mapping, the 1D discrete sensory time-series signal of length *N* needs to be normalized to the interval [−1, 1]. The normalized sequence is denoted as $\tilde{x}$, and its calculation formula is:

(1) $${\tilde{x}}_{i}=\frac{\left(x_{i}-\max(X))+(x_{i}-\min(X)\right)}{\max(X)-\min(X)}$$

Subsequently, the normalized numerical sequence is mapped into a polar coordinate system. The numerical values are encoded as the polar angle, and the corresponding timestamps are encoded as the polar radius:

(2) $$\phi_{i}=\arccos({\tilde{x}}_{i}),\;\phi_{i}\in [0,\pi ]$$

(3) $$r_{i}=\frac{t_{i}}{N}$$

This timestamp-based polar coordinate mapping not only achieves a monotonic bijection, thereby avoiding information loss, but also strictly preserves the absolute temporal dependencies of the signal’s evolution over time.

Finally, to extract the dynamic correlation and spatial synergistic intensity between different time steps, the algorithm utilizes trigonometric expansion to calculate the sum or difference in the polar angles between any two time steps, constructing the Gramian angular summation field (GASF) and the Gramian angular difference field (GADF), respectively. Their mathematical definitions are as follows:

(4) $$GASF_{i,j}=\cos(\phi_{i}+\phi_{j})={\tilde{x}}_{i}{\tilde{x}}_{j}-\sqrt{1-{\tilde{x}}_{i}^{2}}\sqrt{1-{\tilde{x}}_{j}^{2}}$$

(5) $$GADF_{i,j}=\sin(\phi_{i}-\phi_{j})=\sqrt{1-{\tilde{x}}_{i}^{2}}{\tilde{x}}_{j}-{\tilde{x}}_{i}\sqrt{1-{\tilde{x}}_{j}^{2}}$$

In terms of matrix structure, GASF and GADF possess explicit physical and geometric significance. Their main diagonals (or anti-diagonals) contain the temporal evolution trajectory of the original signal, while the remaining matrix elements characterize the spatial correlation and non-linear evolutionary divergence between different time nodes. Through the GAF transformation, the hidden degradation trends within the 1D time-series signal are reconstructed into high-dimensional 2D images with rich textures and grayscale distributions, thereby providing an excellent representational foundation for downstream deep vision models to extract deep degradation features.

### 2.2. OPM

In the dimensional representation of time-series signals, unlike mapping transformations that rely on polar coordinates or probabilistic Markov chains, the Outer Product Matrix (OPM) is an image reconstruction technique that directly calculates spatio-temporal correlations based on the instantaneous amplitude of the signal [21]. Through the outer product operation of vectors, this method can losslessly preserve the absolute amplitude information of the original signal and explicitly quantify the interactive energy intensity between different time nodes or distinct physical features.

For a given one-dimensional discrete sensory feature sequence $X=\{x_{1},x_{2},\ldots ,x_{N}\}$, its self-outer product matrix can be directly defined by the tensor product of the column vector and the row vector:

(6) $$OPM=X⊗X^{T}$$

Any element within the matrix represents the dot product of the signal amplitudes at the *i*-th and *j*-th feature nodes, namely:

(7) $$OPM_{i,j}=x_{i}\cdot x_{j}$$

In the complex scenarios of multi-source heterogeneous sensory monitoring, to extract the synergistic evolution patterns of cross-modal physical features, the Cross Outer Product Matrix is further introduced. Assuming there are two sets of degradation feature sequences, *X* and *Y*, which have been reconstructed according to their physical hierarchy, their cross outer product matrix is calculated as follows:

(8) $$OPM_{cross}=X⊗Y^{T}$$

Its matrix elements, $OPM_{cross(i,j)}=x_{i}\cdot y_{j}$, are capable of characterizing the cross-coupling intensity of heterogeneous signals at different nodes.

### 2.3. CSA-Kansformer

**Figure 1:**
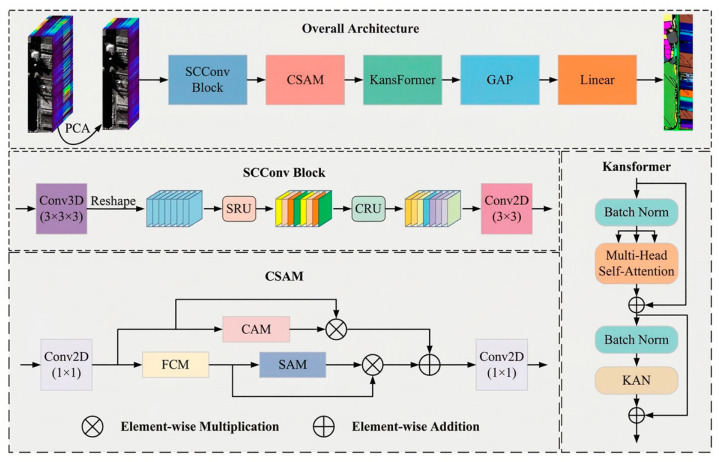
Architecture of the CSA-Kansformer model. The arrows indicate the direction of data flow. Different colors are used to visually distinguish various functional modules, which are explicitly labeled with text.

To break through the bottlenecks of computational redundancy and non-linear fitting inherent in traditional deep networks when processing high-dimensional composite features, this paper introduces the recently proposed CSA-Kansformer deep learning architecture as the classification model for tool wear states [22]. This architecture innovatively integrates local feature extraction with global perception capabilities, and is primarily composed of the spatial and channel reconstruction convolution (SCConv), the cross-scale aggregation module (CSAgM), and the core Kansformer module working in synergy. Its structural schematic is shown in Figure 1.

First, aiming at the feature redundancy prone to occur when high-dimensional fused images are input, the model employs the SCConv module to reduce computational overhead. The spatial redundancy unit (SRU) within this module utilizes the group normalization (GN) scaling factor to evaluate the amount of feature information and extracts core features through a “separate-and-reconstruct” strategy. Its feature normalization formula is as follows:

(9) $$X_{out}=GN(X)=\frac{X-\mu }{\sqrt{\sigma^{2}+\epsilon }}\cdot \gamma +\beta$$

Subsequently, the Channel Redundancy Unit (CRU) further eliminates useless channel mappings through “split–transform–fuse” operations, thereby significantly reducing the number of parameters while preserving critical spatio-temporal features.

Secondly, to enhance the model’s perception and expression capabilities regarding key local features, the CSAM module is introduced for cross-scale aggregation. This module utilizes convolution kernels of different sizes to extract multi-scale information and constructs the channel attention module (CAM) and spatial attention module (SAM) in parallel. CAM captures global channel information and adaptively evaluates weights, while SAM focuses on key spatial high-energy regions along the channel axis. The outputs of the dual attention mechanism and their final deep fusion formula for spatio-temporal features are as follows:

(10) $$X_{CAM}=X⊙\sigma (A_{avg}+A_{max})$$

(11) $$X_{SAM}=X⊙\sigma (Conv2D_{7\times 7}([X_{avg},X_{max}]))$$

(12) $$X_{CSAM}=Conv2D_{1\times 1}(X_{CAM}+X_{SAM})$$

Finally, Kansformer is the core module for processing extreme non-linear degradation features. It utilizes a multi-head self-attention mechanism to capture global dependencies and replaces the layer normalization (LN) in traditional networks with batch normalization (BN) to accelerate convergence and enhance the model’s generalization capability. Its self-attention feature extraction score is calculated as follows:

(13) $$SA=Attention(Q,K,V)=softmax\left(\frac{QK^{T}}{\sqrt{d_{K}}}\right)V$$

Building upon this, the module discards the fixed multi-layer perceptron (MLP) and innovatively introduces the KAN network. KAN utilizes learnable spline functions deployed on the network edges to replace linear weight matrices, achieving precise approximation of high-order non-linear features with extremely high parameter efficiency. Its network mapping calculation is as follows:

(14) $$KAN(x)=(\Psi_{N-1}\circ \Psi_{N-2}\circ \ldots \circ \Psi_{1}\circ \Psi_{0})x\;$$

This fundamental improvement in the mathematical architecture significantly broadens the classification decision boundaries for extreme wear features under complex working conditions.

## 3. PG-MGAF + CSA-Kansformer Tool Wear Status Recognition Model

**Figure 2:**
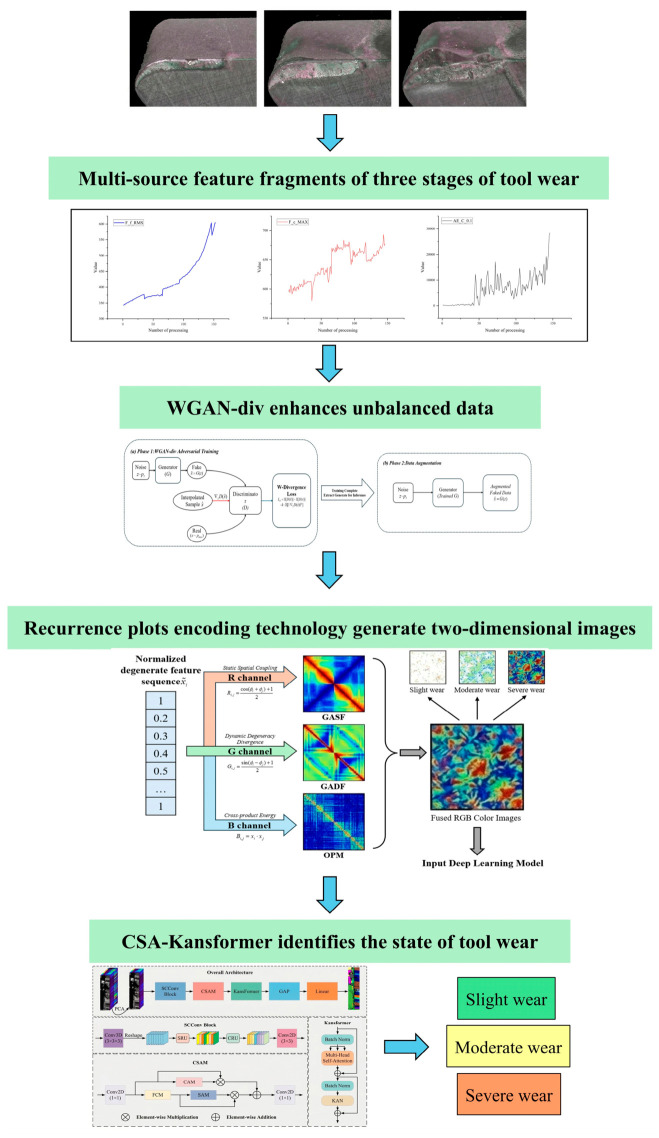
Wear state identification architecture based on PG-MGAF and CSA-Kansformer. The arrows indicate the direction of data flow. Different colors are used to visually distinguish various functional modules, which are explicitly labeled with text.

The proposed method integrates the physics-guided multi-channel feature fusion (PG-MGAF) mechanism with the lightweight hybrid vision architecture, CSA-Kansformer. By utilizing PG-MGAF to convert 1D multi-source sensor time-series signals into 2D high-fidelity color texture images as network inputs, it comprehensively excavates the spatial topological correlations and non-linear degradation features among multi-source signals under different tool wear states. This enables the computer vision network to exhibit significantly stronger responsiveness and feature extraction capabilities for complex mechanical degradation signals compared to traditional 1D sequence models. Meanwhile, leveraging the Channel-Spatial Attention (CSA) mechanism embedded in the CSA-Kansformer and the lightweight computational advantages of KAN, the network inference time is substantially compressed while ensuring high-precision feature extraction, perfectly satisfying the stringent real-time requirements of industrial edge computing. Figure 2. illustrates the specific 1D-to-2D image reconstruction and CSA-Kansformer real-time identification architecture. The specific operational steps of this method can be described as follows:

Step 1: Multi-source signal acquisition, robust preprocessing, and WGAN-DIV-based data augmentation. Multi-source digital signals such as “force–vibration–acoustic emission” are acquired during machine tool operations. First, abnormal extreme value suppression and exponential moving average (EMA) smoothing are applied to the original features to reduce high-frequency mutation interference, and the initial cutting mean value of a brand-new tool is extracted as the dynamic physical baseline for the healthy state. Subsequently, to alleviate the scarcity of samples in the slight-wear and severe-wear stages, a WGAN-DIV-based minority-class augmentation strategy is introduced. In this strategy, Gaussian random noise is used only as the latent input of the generator, while the generated samples are constrained by adversarial divergence learning to approximate the real minority-class feature distribution. This process expands the training samples of critical wear stages without directly perturbing the true wear labels, thereby improving the balance of the training data while preserving the continuity of tool degradation.

Step 2: Global normalization and 2D multi-channel map reconstruction. The global maximum value across the full life cycle is extracted for physical-scale logarithmic normalization to obtain the degradation increment. Following this, by calculating the Gramian angular summation field (GASF), Gramian angular difference field (GADF), and the cross OPM, the 1D sequences are losslessly converted into high-dimensional 2D color maps containing three RGB channels. After assigning physical labels, a leave-one-tool-out (LOTO) cross-validation strategy is adopted to partition independent training and test sets.

Step 3: Minimalist network learning and test time anti-drift calibration. A minimalist CSA-Kansformer network with a depth of only 14 layers is constructed. Mixup data augmentation and asymmetric cross-entropy loss are combined during model training to precisely fit the blurred boundaries of wear transition states. After training is completed, to address the sensor zero-drift phenomenon in industrial sites, a low-overhead test time logit calibration mechanism is introduced during the inference stage for blind testing on the independent set. Finally, it outputs the full life-cycle wear state classification results and the single-sample inference latency.

## 4. Experimental Validation of Tool Wear State Recognition

### 4.1. Data Acquisition and Data Processing

#### 4.1.1. Introduction to Datasets

**Figure 3:**
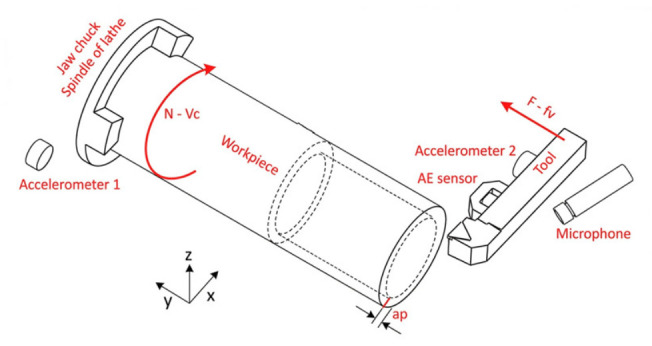
Schematic diagram of the turning process and sensor locations. The curved arrow on the workpiece (labeled N-Vc) indicates the direction of rotation, while the straight arrow on the tool (labeled F-fv) represents the feed direction.

The test data included in this work is sourced from controlled external turning experiments (Vicomtech Turning Tool Wear Dataset) [23]. The specific machining parameters for the turning experiments are presented in Table 1. During the tests, triaxial accelerometers were installed on the lathe spindle and tool holder, respectively, to record cutting vibration signals. Simultaneously, a dynamometer, acoustic emission sensors, and current/voltage sensors were equipped to capture multimodal signals during the machining process. A schematic diagram of the turning process and sensor locations is shown in Figure 3. The experiments utilized P25 uncoated cemented carbide inserts to perform turning operations on 19NiMoCr6 steel workpieces.

**Figure 4:**
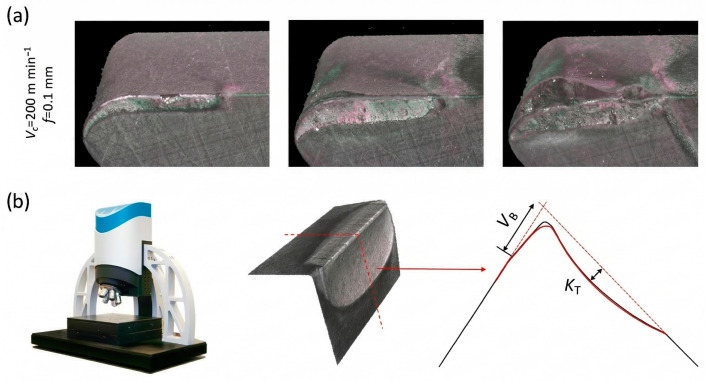
Wear measurement strategy using the Alicona InfiniteFocus G4. (**a**) 3D datasets of tool wear evolution obtained from one repetition using the Alicona Infinite Focus G4; (**b**) extracted mid-plane profile of the contact section used for Vb measurement, with the wear mode indicated.

**Figure 5:**
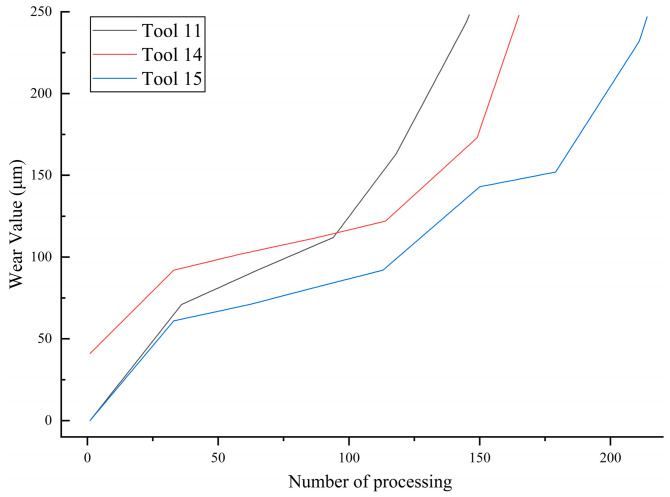
Tool wear curves (Tool 11, Tool 14, and Tool 15).

This study selected the full-life-cycle data of three tools (Tool 11, Tool 14, and Tool 15) from the dataset for subsequent feature transformation and prediction research. During the cutting process, to accurately quantify the tool wear state, periodic offline scanning of the inserts was conducted; the wear measurement strategy using the Alicona InfiniteFocus G4 is illustrated in Figure 4. Flank wear (VB) was calculated by extracting the middle profile of the contact cross-section. According to industrial machining standards, a flank wear of 250 μm on the cutting edge was set as the critical wear threshold for tool failure. The actual wear evolution curves of these three tools with the increasing number of passes are shown in Figure 5. According to the general laws of the tool wear process, this study innovatively divides the tool wear states into three stages based on the proportion of the number of passes over the full life cycle: the first 15% of the total passes is classified as the slight wear region. During this stage, the tool initially contacts the workpiece, and microscopic asperities are rapidly smoothed out, and wear occurs relatively quickly. The middle 70% of passes is defined as the moderate wear region. During this period, the cutting state is relatively stable, the wear land width expands steadily, and the amount of wear exhibits a uniform and slow increase. The final 15% of passes is defined as the severe wear region. At this point, the tool approaches the 250 μm critical failure threshold, cutting conditions deteriorate sharply, cutting temperatures rise, and wear intensity is significantly exacerbated. The specific pass intervals for the three tools across different wear stages, divided based on the proportion of passes, are listed in Table 2.

To fully validate the robustness and generalization ability of the predictive models, a leave-one-tool-out cross-validation strategy was adopted for the three tools (Tool 11, Tool 14, and Tool 15) at the tool level: Experiment A uses Tool 14 and Tool 15 as training samples, with Tool 11 as the test sample; Experiment B uses Tool 11 and Tool 15 as training samples, with Tool 14 as the test sample; and Experiment C uses Tool 11 and Tool 14 as training samples, with Tool 15 as the test sample.

#### 4.1.2. Robust Cleaning and Temporal Distribution Correction of Multi-Source Features for Complex Operating Conditions

Under complex turning conditions, multi-source sensory features are frequently corrupted by extreme pulses and exhibit massive dimensional discrepancies, which can severely disrupt the convergence stability of prediction models. Therefore, this paper proposes a robust multi-source feature preprocessing mechanism, specifically comprising three key steps. First, extreme outlier suppression under strong noise environments is conducted by applying a 1st to 99th percentile clipping to the feature sequences, filtering out transient mutation noise while retaining the core degradation information. Second, a logarithmic affine transformation oriented towards heavy-tailed physical distributions is implemented. Addressing the right-skewed heavy-tailed characteristics exhibited by signals such as acoustic emission (AE) during the severe wear stage, the log1p function is introduced for non-linear smoothing to prevent weak degradation information from being overly compressed. Finally, robust normalization (Robust Scaler) based on the interquartile range is performed. Centered on the median, this step eliminates dimensional differences and smoothly maps the multi-source features into a unified dimensionless space. In addition, exponential moving average (EMA) smoothing is applied to reduce local random fluctuations while preserving the overall degradation tendency of the feature sequences.

**Figure 6:**
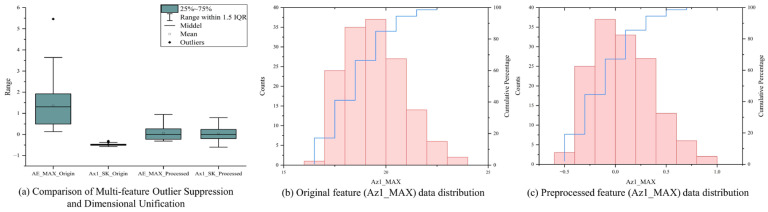
Comparison before and after preprocessing.

The comprehensive effect comparison before and after the aforementioned feature preprocessing is illustrated in Figure 6. Specifically, Figure 6a intuitively demonstrates the global comparison before and after multi-feature outlier suppression and dimensional unification; the originally extremely divergent feature space is highly standardized after processing. To further analyze local details, Figure 6b,c take the maximum *Z*-axis vibration acceleration (Az1_MAX) as an example to clearly present the evolution of the data distribution morphology for a single feature before and after processing. The results indicate that this preprocessing mechanism significantly enhances the signal-to-noise ratio and distribution consistency of the feature matrix, laying a solid data foundation for subsequent high-dimensional physical image reconstruction and high-accuracy tool wear state identification.

It should be noted that the preprocessing pipeline in this study consists of two levels. The first level is the basic robust cleaning stage described in this subsection, including outlier clipping, log1p transformation, robust normalization, and temporal smoothing. The second level is the physics-guided reconstruction stage described in Section 4.1.4, including physical feature reordering and baseline calibration before PG-MGAF image construction. Therefore, the subsequent ablation study evaluates these operations as an integrated data-processing pipeline rather than as isolated preprocessing steps.

#### 4.1.3. Using WGAN-Div to Generate Real-World Data to Overcome Data Imbalance and Statistical Consistency Validation

**Figure 7:**
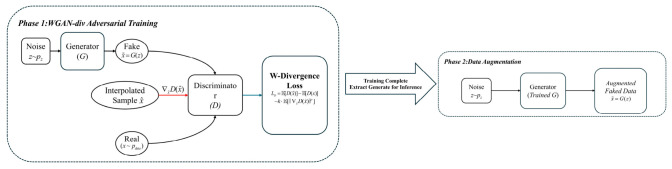
Framework of the WGAN-DIV.

During the full-life-cycle turning operations of cutting tools, according to the wear stages delineated in this study, the feature sample size of the normal wear stage is significantly larger than that of the early and severe wear stages. This typical data class imbalance will lead to a severe recognition bias towards the majority class in deep learning models during training, thereby drastically weakening the model’s generalization accuracy and sensitivity to early weak wear and critical failure states of the tool. To break through this data barrier, this paper introduces a divergence-regularized Wasserstein generative adversarial network (WGAN-DIV) to perform time series data augmentation on minority class samples [24]. The core working framework of WGAN-DIV is shown in Figure 7.

Compared to traditional GAN and WGAN-GP, WGAN-DIV innovatively introduces the Wasserstein divergence, enabling more stable adversarial training without imposing strict Lipschitz continuity constraints (such as weight clipping or gradient penalty) on the discriminator. This equips it with superior convergence and feature fidelity when processing complex, high-frequency physical cutting signals. In the augmentation mechanism of this study, the generator maps Gaussian latent noise into synthetic multi-source feature sequences, while the discriminator evaluates the distribution discrepancy between real and generated features through Wasserstein divergence. Specifically, WGAN-DIV was used to directionally augment the scarce slight-wear and severe-wear samples at an augmentation ratio of 1:5. This operation expanded the minority-class training samples and effectively alleviated the class imbalance in multi-source cutting signal data, thereby encouraging the downstream recognition model to learn weak early-wear features and critical severe-wear features more sufficiently.

**Figure 8:**
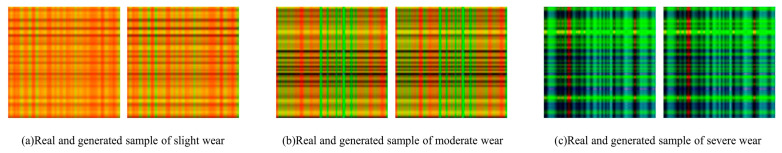
Comparison of reconstructed 2D PG-MGAF images between real and generated samples.

To comprehensively validate the physical fidelity of the data generated by WGAN-DIV, this study conducted a dual comparative analysis, both qualitatively and quantitatively. The PG-MGAF 2D image comparison, reconstructed from real samples and generated samples, is shown in Figure 8.

At the qualitative visual level, comparison of the reconstructed PG-MGAF maps shows that the samples generated by WGAN-DIV retain a high degree of similarity to the real cutting samples in terms of spatial topology, color-evolution tendency, and local high-energy texture distribution. To quantitatively evaluate this fidelity, the Maximum Mean Discrepancy (MMD) in the one-dimensional feature domain and the Structural Similarity Index Measure (SSIM) in the two-dimensional image domain were employed. The MMD value between the real and generated multi-source samples was 0.08, while the average SSIM of the reconstructed PG-MGAF images reached 0.964. These results suggest that the generated samples preserved the main structural characteristics of the original degradation data without introducing obvious distributional distortion.

To further assess the distributional fidelity of the generated data, statistical consistency analysis was additionally performed on representative features from the slight-wear and severe-wear stages. As summarized in Table 3, in the slight-wear stage, more than 90% of the tested features remained non-significant in both the Kolmogorov–Smirnov test and the Mann–Whitney U test, and more than 90% exhibited only small or negligible effect sizes. In the severe-wear stage, over 90% of the tested features remained non-significant in the Kolmogorov–Smirnov test, more than 80% remained non-significant in the Mann–Whitney U test, and more than 90% still showed only small or negligible effects. Overall, these results indicate that most representative features did not exhibit statistically detectable distributional shifts between real and generated samples. Combined with the low MMD and high SSIM values, this finding further supports the distributional fidelity and practical reliability of the WGAN-DIV augmentation strategy for subsequent PG-MGAF reconstruction and deep feature learning.

#### 4.1.4. Physics-Guided Multi-Channel Gramian Angular Field

To break through the spatial dimension limitations of 1D time-series features in representing complex degradation processes, and to fully stimulate the advantages of cutting-edge 2D vision models in high-dimensional texture analysis and local anomaly perception, this paper proposes a physics-guided multi-channel Gramian angular field (PG-MGAF) image reconstruction strategy. This strategy aims to cross-dimensionally transform abstract and discrete 1D multi-source sensory time-series sequences into RGB color maps containing rich degradation mechanisms and high-fidelity energy topologies, thereby injecting strong physical priors into the deep learning model from the data foundation.

Addressing the pain points that multi-source sensory features often exhibit disorder, dimensional isolation, and difficulties in cross-modal interaction, the PG-MGAF algorithm designs a reconstruction mechanism comprising the following three core steps:

1. Physical Sequence Reordering

Aiming at the defect that traditional feature concatenation is prone to producing high-frequency visual tearing in the Gram matrix domain, this algorithm designs a strict sequence reconstruction based on physical mechanisms. Specifically, the algorithm logically rearranges the discrete features according to the sensorial-source physical hierarchy of “cutting force—vibration—acoustic emission.” This rearrangement mechanism establishes a continuous topological correlation path from macroscopic low-frequency (macroscopic tribological evolution reflected by cutting force), to mesoscopic frequency band (structural dynamic response reflected by vibration), and finally to microscopic high-frequency (microscopic material lattice fracture reflected by acoustic emission). This smooth physical gradient transition ensures the topological coherence and deep interpretability of the subsequent mapping to the polar coordinate space and 2D image distribution from the source.

2. Dynamic Baseline Calibration

**Figure 9:**
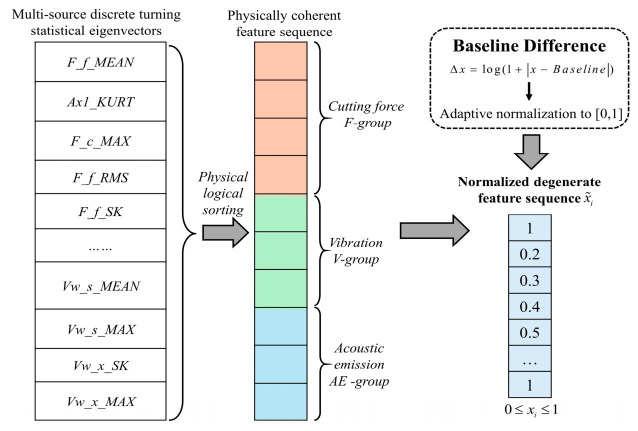
Preprocessing framework of physical rearrangement and baseline calibration for multi-source features.

To acutely capture the feature distortion across the full life cycle of the cutting tool, this paper extracts the local feature mean value of a brand-new cutting tool at the initial cutting stage as the system’s “health baseline.” Subsequently, the logarithmic absolute deviation between the currently monitored features and the health baseline is calculated. The dual physical significance of introducing the logarithmic affine transformation lies in the fact that, in the early degradation stage, it can effectively amplify weak feature variations, while in the severe wear stage at the end of the life cycle, it serves as a smooth buffer to effectively suppress exponentially erupting extreme fluctuations. Next, utilizing the global maximum values extracted within the full life cycle of the tool, the aforementioned deviation sequences are adaptively normalized to the [0, 1] interval. This process completely eliminates the dimensional barriers of multi-source heterogeneous signals, yielding a set of standardized sequences capable of accurately characterizing the absolute degradation increment. The preprocessing framework for the aforementioned physical logical rearrangement and dynamic baseline calibration of multi-source features is illustrated in Figure 9.

3. Multi-Channel Heterogeneous Fusion

**Figure 10:**
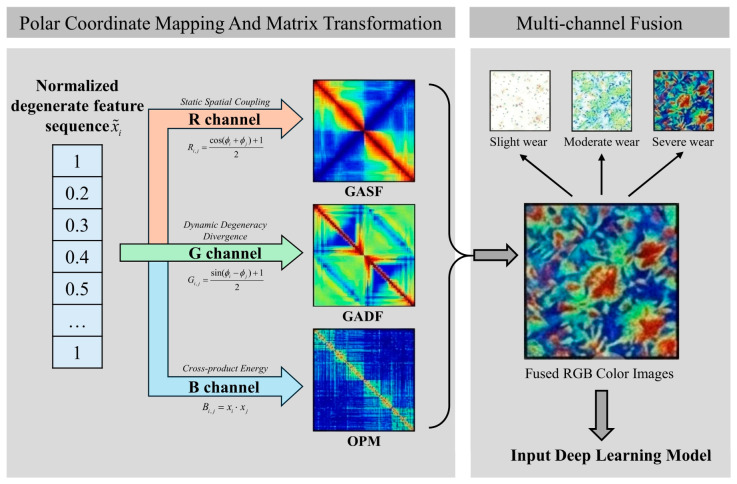
Framework of the PG-MGAF.

To further construct a wear state representation with extremely high discriminability, this paper breaks through the expression limitations of traditional grayscale images or single-channel time-frequency maps, which are prone to losing cross-modal information. The algorithm losslessly maps the normalized sequences to the polar coordinate system via the arccosine function, preserving the absolute temporal dependencies and relative angular topology of the features. Subsequently, three spatial matrix transformation methods of different dimensions are respectively injected into the three RGB color channels to construct a deeply fused pseudo-color image. Its overall framework is shown in Figure 10:

R Channel (static spatial coupling): Calculated using the Gramian angular summation field (GASF). Utilizing the sum angle formula, this channel maps the polar coordinate time series into an inner product spatial matrix, completely preserving the static interdependencies and global spatial synergistic intensity among multi-source physical features, constituting a coherent background foundation texture.

G Channel (dynamic degradation divergence): Calculated using the Gramian angular difference field (GADF). Through the difference angle operation, this channel is extremely sensitive to fluctuations of adjacent sequences and cross-modal boundaries. It can acutely amplify the non-linear evolution divergence of the “force–vibration–acoustic emission” transition zone during the degradation process, intuitively highlighting the dynamic mutation edges when wear exacerbates.

B Channel (cross outer product energy): The cross OPM of the original 1D features is innovatively introduced as an energy compensation term. This channel bypasses the non-linear mapping of trigonometric functions and directly reflects the interactive multiplier and absolute physical energy level between different physical features. It preserves the most primitive signal amplitude correlations, providing a solid energy base for multi-channel fusion.

The aforementioned three matrices achieve perfect complementarity in mathematical mechanism and physical mapping. After deep concatenation (Concat) at the channel level, a high-dimensional tensor simultaneously accounting for “static topology, dynamic divergence, and absolute energy” is generated. When this fused map, imbued with rich prior physical knowledge, is fed into the downstream network, it can simultaneously provide highly discriminative high-order representations for the model in both the spatial receptive field and deep channel dimensions, thereby greatly enhancing the model’s robustness against local signal distortion and environmental noise.

**Figure 11:**
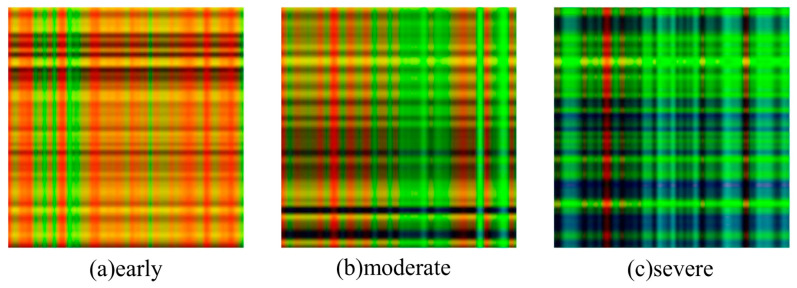
Multi-channel fused RGB images corresponding to different wear stages of the cutting tool.

As shown in the visualization results in Figure 11, with the continuous exacerbation of the tool wear state, the color patches representing high-frequency fluctuations and high-energy interactions in the fused map exhibit a significant regular evolution: not only does the area of bright (high-energy) color patches expand outward, but the high-response regions also gradually gather towards the main diagonal. This visualized topological evolution law not only intuitively reflects the physical essence of tool degradation but also effectively widens the non-linear decision boundary of the downstream deep learning identification model when extracting extreme wear or complex micro-fracture features.

### 4.2. Experimental Results and Analysis

#### 4.2.1. Verifying the PG-MGAF + CSA-Kansformer Model

This study transforms the multi-source 1D monitoring signals across the full life cycle of cutting tools into high-fidelity 2D RGB color texture images via the “physical logical rearrangement-multidimensional feature fusion” strategy, serving as the input for the proposed CSA-Kansformer model. This model innovatively integrates SCConv, CSAM, and the Transformer module embedded with KAN, effectively breaking through the performance bottleneck caused by the weak non-linear representation of traditional deep networks. It significantly enhances the recognition accuracy of wear states under complex working conditions while maintaining low computational cost and inference latency.

The core hyperparameters for this experiment are set as follows: the initial learning rate is 0.0001, and a cosine annealing strategy is employed for dynamic smooth decay during the iteration process. The number of network epochs is set to 100. AdamW (with a weight decay coefficient of) is selected as the optimizer. While inheriting the strong adaptive computational efficiency of Adam, it effectively bolsters the regularization effect on network weights. Furthermore, to enhance the model’s feature extraction and generalization capabilities across different tools, the Mixup mechanism with a hyperparameter is introduced during training.

The loss function of this model utilizes the weighted cross-entropy loss, introducing asymmetric penalty weights to address the sample imbalance across different wear stages and the disparity in misclassification costs [25]. Its mathematical expression is:

(15) $$L=-\frac{1}{N}\sum_{i=1}^{N}\sum_{c=1}^{C}w_{c}\cdot y_{i,c}\log(p_{i,c})\;$$

where *N* is the number of samples in a single training batch; *C* is the total number of wear state classes (*C* = 3 in this study); $w_{c}$ is the asymmetric weight assigned to the *c*-th class. For slight, moderate, and severe wear, these weights are set to 1.2, 1.0, and 2.5, respectively, to deliberately intensify the penalty for misclassifying the severe wear state; $y_{i,c}$ is the indicator function, which equals 1 if the true class of sample *i* is *c*, and 0 otherwise; and $p_{i,c}$ represents the predicted probability output by the network that sample *i* belongs to class *c*. A smaller loss value indicates a superior fit of the model to the true feature distribution, signifying better classification performance.

**Figure 12:**
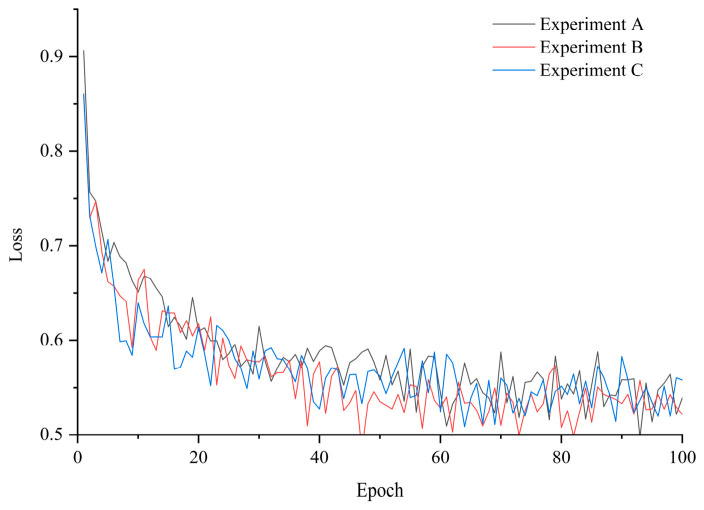
Loss curves of Tool 11, Tool 14, and Tool 15.

To comprehensively evaluate and guarantee the true generalization performance of the model, this paper strictly adopts the LOTO cross-validation strategy to conduct three sets of experiments (Experiments A to C). The loss reduction curves of the model during the training process are shown in Figure 12. As depicted in the figure, the CSA-Kansformer model exhibits a rapid convergence speed, and the entire descending process is characterized by minor fluctuations and a stable trend. This indicates that the network possesses excellent learning efficiency and convergence robustness during the training phase.

**Figure 13:**
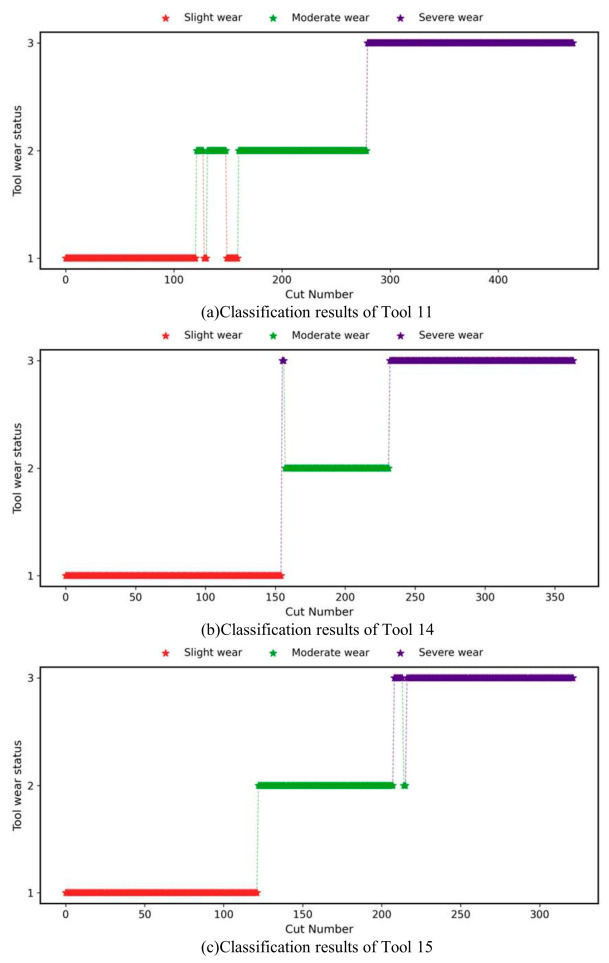
Recognition results of the CSA-Kansformer for the three tools.

Figure 13 displays the recognition and classification results of the CSA-Kansformer model for Experiments A through C. From left to right, the red, green, and purple lines represent slight wear, moderate wear, and severe wear, respectively. Asterisks deviating from the main step line indicate misclassifications. Out of the 322 samples in Experiment A, only 15 samples were misidentified. Notably, no cross-level recognition errors were observed in the severe wear stage; 3 samples misclassified severe wear as moderate wear, and 12 samples misclassified moderate wear as slight wear. In Experiment B, 29 out of 364 samples were misidentified. There were no recognition errors in the slight wear stage. The misjudgments were mainly concentrated in the transition phase from moderate wear to severe wear, including prematurely warning moderate wear as severe wear and conservatively identifying a small portion of severe wear as moderate wear. Such misjudgments have a relatively minor negative impact on actual machining decisions. In Experiment C, 28 samples were misjudged. While there were absolutely no errors in the severe wear stage, some samples with moderate wear were misclassified as severe wear. This tends to cause premature scrapping and replacement of the tool before its useful life ends, which warrants attention.

**Figure 14:**
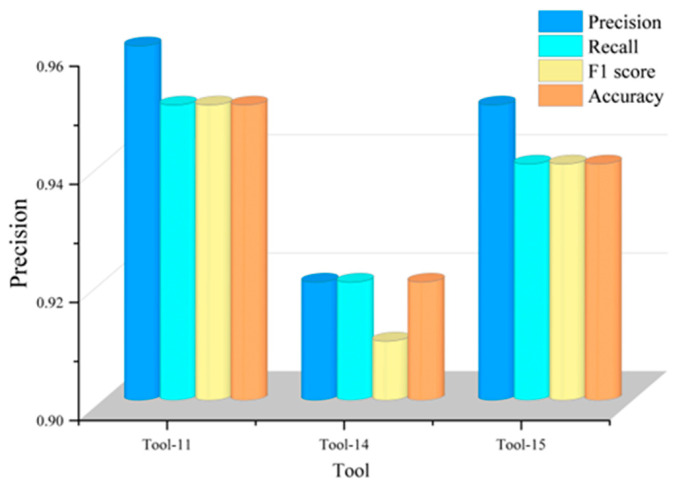
Visualization of performance evaluation metrics for the three tools.

To further quantitatively analyze the generalization performance of the CSA-Kansformer model and enhance persuasiveness, we visualized the core performance scoring metrics of the multiple LOTO cross-validation experiments, with the results shown in Figure 14. In the cross-experiments targeting Tools 11, 14, and 15, the overall accuracies of the model were 0.95, 0.92, and 0.94, respectively; the Macro F1 scores were 0.95, 0.92, and 0.94, respectively; and the independent recall rates for the “severe wear” category reached 0.97, 0.95, and 0.94, respectively. Integrating all LOTO cross-validation experiments, the average accuracy of the model reaches 0.936, and the single-sample average inference latency is only 5.90 ms. All evaluation metrics are maintained at a high level, fully demonstrating that the CSA-Kansformer model possesses excellent stability, noise-resistant robustness, and cross-tool generalization performance for the tool wear state identification task under the present turning setup.

The exceptional performance achieved by the proposed combined model under complex cutting conditions is attributed not only to its lightweight network architecture but also to the deep interpretability alignment between its internal mechanisms and the proposed high-dimensional physical images. It is particularly noteworthy that, unlike traditional general-purpose attention mechanisms designed directly for natural images, the CAM in the proposed model is endowed with a clear physical representation orientation. Because the input preprocessed tensor is strictly physically encoded along the channel dimension—where the R channel represents the static spatial coupling among multi-source signals (GASF), the G channel signifies the dynamic evolutionary divergence of the degradation process (GADF), and the B channel directly maps the absolute interactive energy between features (the cross OPM)—the weight vector output by the CAM essentially evaluates the absolute contribution of these three heterogeneous physical attributes to the current local wear state dynamically. During the actual physical machining process, this mechanism plays a critical role: for instance, in the stable phase of initial tool wear, the network adaptively assigns a higher activation weight to the R channel; conversely, during the mutation period transitioning from moderate to severe wear, the volatility of adjacent cutting features rises sharply, at which point the CAM keenly captures this variation and substantially elevates the weight distributions of the G channel and the B channel. Through this data-driven channel reallocation mechanism, the proposed model successfully overcomes the limitations of blind feature searching inherent in deep learning, achieving an adaptive focus on the key physical feature flows strongly correlated with the actual tool degradation mechanisms.

#### 4.2.2. Progressive Ablation Study of the Preprocessing Pipeline

To quantitatively evaluate the marginal contribution of the complete data-processing pipeline, a progressive ablation study was conducted under the same LOTO cross-validation protocol and classifier configuration. The evaluated pipeline covers both the basic robust preprocessing operations described in Section 4.1.2 and the physics-guided reconstruction operations described in Section 4.1.4. Starting from a minimal input pipeline (S0), clipping, log1p transformation, RobustScaler normalization, physical reordering, EMA smoothing, and baseline calibration were sequentially introduced to construct six progressively enhanced preprocessing settings (S1–S6). The corresponding results are summarized in Table 4.

As shown in Table 4, the classification performance improved steadily as additional preprocessing components were incorporated. The raw/minimal pipeline (S0) achieved only 0.7135 in mean accuracy, 0.4941 in mean Macro-F1, and 0.5023 in mean severe-wear recall, indicating that the original multi-source features were still strongly affected by impulsive noise, heavy-tailed distributions, dimensional inconsistency, and weak inter-sensor coupling. After clipping and log1p transformation were introduced (S1–S2), the performance improved only slightly, suggesting that these two operations mainly played a stabilizing role by suppressing extreme fluctuations and reducing heavy-tail distortion.

A more substantial improvement was observed after RobustScaler normalization was added in S3, where the mean accuracy increased to 0.8010, the mean Macro-F1 rose to 0.6420, and the mean severe-wear recall reached 0.6620. This result indicates that dimensional alignment across heterogeneous sensors is a necessary prerequisite for stable feature interaction. When physical reordering was further introduced in S4, the three metrics increased to 0.8562, 0.7760, and 0.7612, respectively, showing that arranging the multi-source features according to the force–vibration–acoustic emission hierarchy helped the model better capture cross-modal degradation continuity.

The subsequent addition of EMA smoothing in S5 further improved the mean accuracy to 0.9042 and the mean severe-wear recall to 0.8823, demonstrating that temporal smoothing effectively reduced local fluctuation interference while preserving the overall degradation trend. Finally, after baseline calibration was incorporated in S6, the complete preprocessing pipeline achieved the best overall performance, with a mean accuracy of 0.9364, a mean Macro-F1 of 0.9372, and a mean severe-wear recall of 0.9543. Compared with S0, the full pipeline improved these three metrics by 22.29, 44.31, and 45.20 percentage points, respectively.

Overall, the progressive improvement from S0 to S6 indicates that the proposed preprocessing pipeline is effective not only as a noise-robust data cleaning strategy, but also as a physics-guided representation enhancement mechanism. In particular, the larger gains brought by RobustScaler normalization, physical reordering, EMA smoothing, and baseline calibration suggest that the final performance improvement mainly comes from restoring inter-sensor comparability, reinforcing degradation continuity, and highlighting wear-related increments, rather than from simple signal denoising alone.

#### 4.2.3. Sensitivity Analysis of Weighted Cross-Entropy Loss

To evaluate the robustness of the weighted cross-entropy design, a sensitivity analysis was conducted by varying the class weights assigned to the slight-wear and severe-wear categories, while keeping the moderate-wear class fixed at 1.0. The corresponding results are summarized in Table 5.

As shown in Table 5, the model performance was clearly influenced by the class-weight configuration. Among all tested combinations, the setting of slight/moderate/severe = 1.2/1.0/2.5 achieved the best overall balance, yielding a mean accuracy of 0.9360, a mean Macro-F1 of 0.9370, and a mean severe-wear recall of 0.9540. This result indicates that the weight configuration adopted in this study is not arbitrary, but represents an effective compromise between global classification performance and the safety-critical recognition of severe wear.

A detailed comparison further reveals that moderate enhancement of the minority and high-risk classes is beneficial, whereas excessively aggressive penalization degrades the overall class balance. When the severe-wear weight was set to 2.0, increasing the slight-wear weight from 1.0 to 1.5 gradually improved the mean accuracy from 0.9060 to 0.9172 and the mean severe-wear recall from 0.9113 to 0.9373, suggesting that a proper increase in slight-wear emphasis helps the model better preserve the early-stage degradation boundary. However, when the severe-wear weight was further increased to 3.0, the overall performance dropped markedly across all tested slight-wear weights. For example, under the slight-wear weight of 1.2, the mean accuracy decreased from 0.9360 to 0.8681 and the mean Macro-F1 decreased from 0.9370 to 0.8643 when the severe-wear weight increased from 2.5 to 3.0. This phenomenon suggests that overly large severe-wear penalties may induce over-compensation during optimization, thereby weakening the discrimination of other wear stages and reducing the global stability of the classifier.

Overall, the results demonstrate that the proposed weighted cross-entropy loss is reasonably robust within a moderate parameter range, and that the selected configuration of 1.2/1.0/2.5 provides the most balanced trade-off among overall accuracy, inter-class balance, and severe-wear sensitivity under the present LOTO evaluation setting.

#### 4.2.4. Comparison of Performance Analysis of Different Deep Learning Models

**Figure 15:**
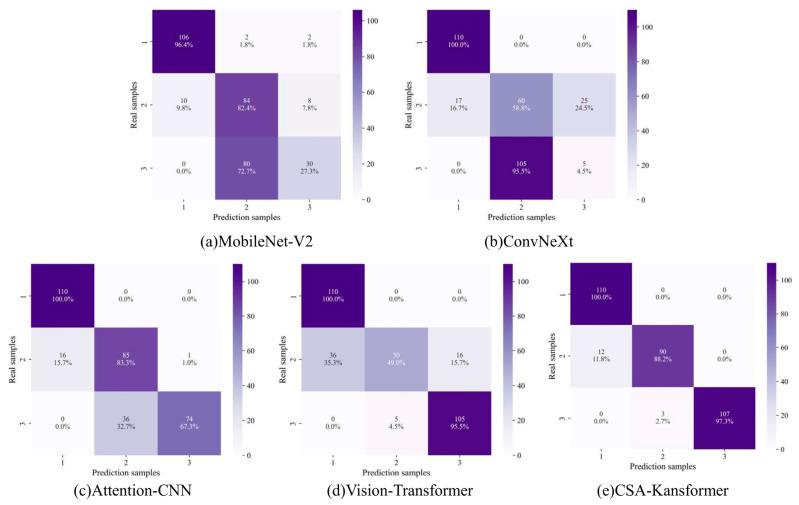
Comparison of confusion matrices among different models.

To further validate the comprehensive performance of different deep learning methods on tool wear state identification, this study introduced four current mainstream deep learning networks as baseline models for a lateral comparison in Experiment C (targeting Tool 15 as the test set). These include: a lightweight convolutional network (MobileNet-V2), a modern pure convolutional architecture (ConvNeXt), a convolutional network integrated with an attention mechanism (Attention-CNN), and a vision backbone network based on global self-attention (Vision-Transformer). The tool wear state identification results and confusion matrix visualizations of the different models are shown in Figure 15.

Through comparative analysis, it can be observed that the misclassifications of different models are mainly concentrated around the moderate wear stage. This phenomenon is reasonable because tool degradation is a continuous physical process, and the moderate wear stage serves as a transition region between slight wear and severe wear. Samples in this stage may therefore contain overlapping degradation characteristics, which increases the difficulty of precise boundary discrimination. For practical turning operations, however, the reliable identification of severe wear is particularly important because missed recognition of critical wear may lead to delayed tool replacement, degraded workpiece quality, or potential damage to machining equipment.

In this regard, the proposed CSA-Kansformer model showed a more stable recognition performance. Among the 470 test samples, only 28 samples were misclassified, and these errors mainly occurred in the transitional region with ambiguous wear characteristics. More importantly, all 160 severe-wear samples in this test fold were correctly identified, corresponding to a severe-wear recall of 100.0%, while the overall accuracy reached 94.0%. These results indicate that the proposed model can maintain high overall classification accuracy while preserving strong sensitivity to safety-critical severe wear. Therefore, compared with the baseline models, CSA-Kansformer provides a more reliable recognition framework for multi-source heterogeneous cutting signals under the present experimental setting.

To intuitively quantify and comprehensively evaluate the classification performance and computational overhead of each model, multi-dimensional evaluation metrics are detailed in Table 6. In terms of classification accuracy, some general vision models exhibited obvious limitations: due to the lack of specific inductive biases for 1D reconstructed images, the accuracies of ConvNeXt and Vision-Transformer fell short of expectations, reaching only 54.0% and 66.0%, respectively. In contrast, relying on multi-scale time-frequency feature aggregation and high-order non-linear representation capabilities, the proposed CSA-Kansformer model demonstrates significant advantages across four core evaluation metrics: Accuracy, Precision, Recall, and F1-score. Specifically, compared to MobileNet-V2, the proposed model improved by 15.0, 16.3, 15.4, and 15.7 percentage points, respectively; compared to ConvNeXt, it substantially increased by 40.0, 14.0, 40.0, and 43.0 percentage points; compared to Attention-CNN, it improved by 4.0, 7.0, 7.0, and 8.0 percentage points; and compared to Vision-Transformer, it exceeded by 28.0, 23.7, 28.4, and 30.0 percentage points, respectively. This indicates that the proposed model can maintain more balanced and competitive classification performance across all stages of tool degradation.

In addition to pursuing high accuracy, real-time online condition monitoring also heavily relies on lightweight network architectures and reasonable computational overhead. The test results in Table 6 further indicate that although MobileNet-V2 and Attention-CNN possess a certain degree of classification accuracy, due to their reliance on the stacking of massive residual modules or complex self-attention computations, their network depths reach 53 and 50 layers, respectively, with single-step inference latencies increasing to 5.89 ms and 6.69 ms. To break through this computational bottleneck, the proposed model introduces SCConv to efficiently eliminate redundant noise, and utilizes the powerful single-layer non-linear approximation capability of KAN to replace the traditional MLP, successfully compressing the network depth significantly to a mere 14 layers. While achieving extreme simplification of the network structure, the single-step inference latency of this model is stably controlled at 5.90 ms.

#### 4.2.5. Influences of Different Signal Processing Methods on Recognition Effect

**Figure 16:**
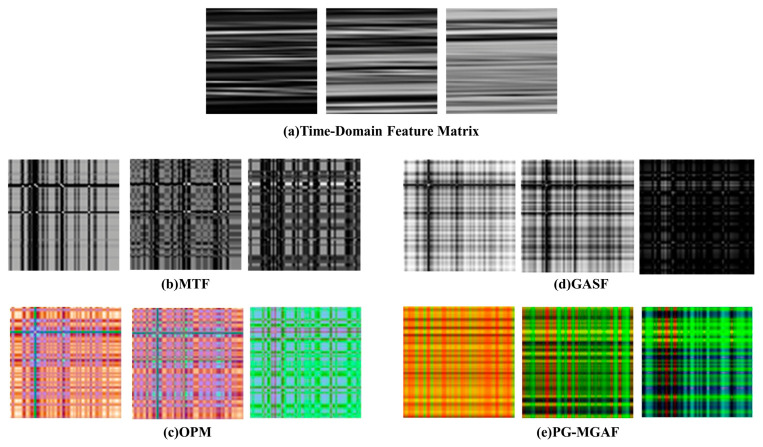
Visual comparison of 2D feature representations generated by different transformation paradigms across three wear stages.

In tool wear state monitoring, transforming one-dimensional multi-source signals into two-dimensional images can provide a more expressive input space for vision-based deep networks. To examine the influence of different signal representation strategies on the final recognition performance, five image construction methods were compared under the same network configuration and the same Tool 15 test setting: time-domain feature mapping (TDFM), MTF, OPM, GASF, and the proposed physics-guided multi-channel Gramian angular field (PG-MGAF). The quantitative results are listed in Table 7, and the visual comparison of different 2D representations is shown in Figure 16.

As shown in Table 7, different imaging strategies exhibit clear differences in their ability to describe tool degradation. TDFM obtained an accuracy of only 0.59 and a Macro-F1 of 0.59. Although its recall for slight and severe wear reached 1.00, its severe-wear precision was only 0.20, indicating that many non-severe samples were incorrectly identified as severe wear. This suggests that simple time-domain mapping can enhance local response regions, but it lacks sufficient discriminative constraints for distinguishing transitional wear states. MTF achieved an accuracy of 0.72, but its Macro-F1 was only 0.50. This is mainly because MTF describes state transition probabilities after discretization, which may weaken the continuous amplitude evolution that is important for tool wear diagnosis. OPM also achieved an accuracy of 0.72, but the recall and F1-score for severe wear were only 0.15 and 0.13, respectively, indicating that amplitude-based interaction alone is insufficient to capture the dynamic transition characteristics of severe degradation.

Among the single-mechanism methods, GASF showed the strongest overall performance, with an accuracy of 0.90 and a Macro-F1 of 0.71. This result indicates that GASF can effectively preserve the global correlation structure and static spatial coupling of the degradation sequence. However, its severe-wear recall was only 0.15, showing that a single static coupling representation is still insensitive to abrupt dynamic changes in the late wear stage. In contrast, the proposed PG-MGAF achieved the best comprehensive performance, with an accuracy of 0.94, a Macro-Precision of 0.95, a Macro-Recall of 0.94, and a Macro-F1 of 0.94. In particular, the severe-wear recall reached 1.00 and the severe-wear F1-score reached 0.92, demonstrating its stronger capability in identifying safety-critical wear states.

The superiority of PG-MGAF comes from its multi-channel complementary representation rather than from a single imaging mechanism. Specifically, GASF provides the static topological structure of the degradation process, GADF enhances the dynamic divergence and transition sensitivity, and OPM preserves the amplitude-dependent interaction energy between features. By integrating these three components into independent RGB channels after physical feature reordering and baseline calibration, PG-MGAF simultaneously retains structural continuity, dynamic variation, and energy interaction information. Therefore, compared with TDFM, MTF, OPM, and GASF, the proposed representation provides a more complete and discriminative description of multi-source tool wear signals, thereby improving both the overall classification performance and the recognition reliability of severe wear.

#### 4.2.6. Ablation Study on RGB Channel Assignment Strategies

Although the PG-MGAF representation proposed in Section 4.1.4 is constructed based on physical interpretation, the rationality of the specific RGB channel assignment still requires quantitative verification. In the proposed framework, GASF is used to describe the static spatial coupling among multi-source degradation features, GADF is used to characterize the dynamic divergence during wear evolution, and OPM is introduced to preserve the inter-feature energy interaction. Therefore, the adopted mapping of R = GASF, G = GADF, and B = OPM is designed to organize the reconstructed RGB image into three complementary information layers: structural continuity, transition sensitivity, and energy compensation. To verify whether this physically guided arrangement is also suitable from a classification perspective, all six possible permutations of GASF, GADF, and OPM were evaluated under the same LOTO cross-validation protocol and classifier configuration.

As shown in Table 8, M1, corresponding to R = GASF, G = GADF, and B = OPM, achieved the best overall performance balance among all tested strategies. Specifically, M1 obtained an average Accuracy of 0.936, an average Macro-F1 of 0.937, and an average severe-wear Recall of 0.954. Compared with M1, the other five permutations led to decreases of 2.7–7.8 percentage points in Accuracy and 3.3–8.6 percentage points in Macro-F1. These results demonstrate that the proposed RGB organization is not an arbitrary color arrangement, but a quantitatively supported fusion strategy.

It is also worth noting that M6, corresponding to R = OPM, G = GADF, and B = GASF, achieved the highest severe-wear Recall of 0.973. However, its average Accuracy and Macro-F1 decreased to 0.909 and 0.904, respectively. This indicates that emphasizing energy interaction in the R channel may further increase the sensitivity to severe wear, but it also weakens the global discrimination ability and inter-class balance of the model. Therefore, severe-wear recall alone is insufficient to determine the optimal RGB mapping strategy; the overall balance among Accuracy, Macro-F1, and severe-wear Recall should be considered simultaneously.

The advantage of M1 is consistent with the complementary roles of the three encoded components. GASF mainly represents the global and relatively stable correlation structure of the degradation process, and placing it in the R channel helps establish a stable structural background for the fused image. GADF is more sensitive to local changes and transition boundaries, and assigning it to the G channel helps highlight the dynamic differences associated with wear evolution. OPM directly retains amplitude-dependent inter-feature energy interactions; when it is assigned to the B channel, it provides an effective energy supplement without disturbing the structural and differential information carried by GASF and GADF. In this way, the final RGB representation forms a coordinated distribution of static topology, dynamic divergence, and interaction energy.

Overall, the ablation results demonstrate that M1 is not an empirical or arbitrary channel assignment. Instead, it provides the most balanced fusion strategy among the six tested permutations and is supported by both physical interpretation and quantitative evidence. Therefore, the mapping of R = GASF, G = GADF, and B = OPM was adopted in all subsequent PG-MGAF reconstruction experiments.

## 5. Conclusions

To address the challenges of insufficient multi-source signal fusion, difficult extraction of non-linear degradation boundaries, and class imbalance in tool wear state identification under complex turning conditions, this study proposed an online tool wear monitoring framework that integrates physics-guided feature image reconstruction with a lightweight CSA-Kansformer architecture. The proposed framework combines WGAN-DIV-based minority-class augmentation, PG-MGAF multi-channel image reconstruction, and a compact deep learning classifier to improve both recognition accuracy and computational efficiency. The main conclusions are summarized as follows:

1. A WGAN-DIV-based minority-class augmentation and PG-MGAF reconstruction strategy was developed for multi-source turning signals. To alleviate the imbalance among wear stages, a divergence-regularized Wasserstein generative adversarial network was used to generate supplementary samples for the slight-wear and severe-wear stages. The distributional fidelity of the generated samples was evaluated using MMD, SSIM, and statistical consistency tests, providing additional evidence for their applicability in subsequent model training. Meanwhile, the one-dimensional multi-source features were rearranged according to their physical hierarchy, and GASF, GADF, and cross OPM were mapped to the R, G, and B channels, respectively. This PG-MGAF representation integrates static coupling, dynamic divergence, and energy interaction information, thereby improving the physical coherence and discriminative capacity of the model input.
2. The CSAM module was endowed with improved physical interpretability through the proposed RGB physical encoding strategy. Unlike general visual attention mechanisms, the CAM in the proposed model can adaptively reallocate weights among the R, G, and B channels, which correspond to static spatial coupling, dynamic degradation divergence, and absolute energy interaction, respectively. This mechanism enables the model to focus on different degradation-related feature flows at different wear stages, thereby enhancing its sensitivity to critical wear characteristics while maintaining a physically interpretable feature selection process.
3. A lightweight CSA-Kansformer network was developed to balance non-linear representation capability and inference efficiency. By introducing SCConv to reduce spatial and channel redundancy and using the KAN module to replace the conventional MLP structure in Transformer-like architectures, the model improves the approximation of high-order non-linear degradation features with a compact network design. The resulting architecture contains only 14 layers, and the single-sample inference latency is controlled at 5.90 ms, indicating its potential for real-time tool condition monitoring.
4. The proposed framework achieved stable cross-tool recognition performance on a real full-life-cycle turning dataset. Under the LOTO cross-validation protocol, the model obtained an average accuracy of 93.6% across three tools. In the Tool 15 test fold, the proposed method achieved an overall accuracy of 94.0% and correctly identified all severe-wear samples, showing strong sensitivity to safety-critical wear states. Compared with mainstream deep learning models and single-mechanism image representation methods, the proposed PG-MGAF + CSA-Kansformer framework achieved a more balanced trade-off among classification accuracy, severe-wear recall, model depth, and inference latency.

Although the proposed framework achieved stable performance under the LOTO protocol using three full-life-cycle tools, the present validation was still conducted within the same turning setup. Cross-condition generalization under varying cutting speeds, feeds, depths of cut, and workpiece materials will be further investigated in future work.

## Figures and Tables

**Table 1:** Machining parameters of the turning experiments.

Cutting Speed (m/min)	Feed Rate (mm/rev)	Depth of Cut (mm)	Machining Length per Pass (mm)
200	0.1	2	70

**Table 2:** Classification of tool wear states.

Cutting Tool	Slight Wear Region	Moderate Wear Region	Severe Wear Region
Tool 11	1–22	23–124	125–146
Tool 14	1–25	26–139	140–165
Tool 15	1–32	33–182	183–214

**Table 3:** Summary of statistical consistency between real and WGAN-DIV-generated samples across representative features.

Stage	Features Tested	KS Non-Significant	Mann–Whitney U Non-Significant	Small/Negligible Effect Size
Slight wear	12	11/12 (91.7%)	11/12 (91.7%)	11/12 (91.7%)
Severe wear	12	11/12 (91.7%)	10/12 (83.3%)	11/12 (91.7%)

**Table 4:** Progressive ablation results of the preprocessing pipeline.

Setting	Clipping	Log1p	RobustScaler	Reordering	EMA	Baseline Calibration	Mean Accuracy	Mean Macro-F1	Mean Severe Recall
S0	×	×	×	×	×	×	0.714	0.494	0.502
S1 (+Clipping)	√	×	×	×	×	×	0.732	0.500	0.518
S2 (+log1p)	√	√	×	×	×	×	0.756	0.504	0.530
S3 (+RobustScaler)	√	√	√	×	×	×	0.801	0.642	0.662
S4 (+Reordering)	√	√	√	√	×	×	0.856	0.776	0.761
S5 (+EMA)	√	√	√	√	√	×	0.904	0.845	0.882
S6 (+Baseline calibration)	√	√	√	√	√	√	0.936	0.937	0.954

**Table 5:** Sensitivity analysis results of the weighted cross-entropy loss under different class-weight settings.

Slight Wear	Moderate Wear	Severe Wear	Mean Accuracy	Mean Macro-F1	Mean Severe Recall
1.0	1.0	2.0	0.906	0.903	0.911
1.0	1.0	2.5	0.873	0.870	0.800
1.0	1.0	3.0	0.871	0.867	0.841
1.2	1.0	2.0	0.912	0.910	0.915
1.2	1.0	2.5	0.936	0.937	0.954
1.2	1.0	3.0	0.868	0.864	0.832
1.5	1.0	2.0	0.917	0.915	0.937
1.5	1.0	2.5	0.891	0.888	0.904
1.5	1.0	3.0	0.891	0.886	0.909

**Table 6:** Performance metrics of different models.

Model		Performance Assessment Indicators					
		Precision	Recall	F1 Score	Accuracy	Depth	Latency (ms)
MobileNet-V2	Slight wear	0.90	0.86	0.88	0.79	53	5.89
	Moderate wear	0.71	0.63	0.66			
	Severe wear	0.75	0.86	0.80			
	Macro Avg	0.79	0.78	0.78			
ConvNeXt	Slight wear	1.00	0.34	0.50	0.54	18	3.95
	Moderate wear	1.00	0.27	0.42			
	Severe wear	0.43	1.00	0.60			
	Macro Avg	0.81	0.54	0.51			
Attention-CNN	Slight wear	0.97	0.92	0.94	0.90	50	6.69
	Moderate wear	0.73	0.95	0.82			
	Severe wear	0.95	0.74	0.83			
	Macro Avg	0.88	0.87	0.86			
Vision-Transformer	Slight wear	1.00	0.45	0.62	0.66	6	1.98
	Moderate wear	0.46	0.51	0.48			
	Severe wear	0.68	1.00	0.81			
	Macro Avg	0.71	0.65	0.64			
CSA-Kansformer	Slight wear	0.99	1.00	0.99	0.94	14	5.90
	Moderate wear	1.00	0.81	0.90			
	Severe wear	0.86	1.00	0.92			
	Macro Avg	0.95	0.94	0.94			

**Table 7:** Performance metrics of five image processing methods.

Methods		Performance Assessment Indicators			
		Precision	Recall	F1 Score	Accuracy
TDFM	Slight wear	0.78	1.00	0.88	0.59
	Moderate wear	1.00	0.38	0.56	
	Severe wear	0.20	1.00	0.33	
	Macro Avg	0.66	0.79	0.59	
MTF	Slight wear	0.83	0.07	0.13	0.72
	Moderate wear	0.77	0.98	0.86	
	Severe wear	0.42	0.70	0.52	
	Macro Avg	0.67	0.58	0.50	
OPM	Slight wear	1.00	0.54	0.70	0.72
	Moderate wear	0.75	0.86	0.80	
	Severe wear	0.12	0.15	0.13	
	Macro Avg	0.62	0.52	0.54	
GASF	Slight wear	0.94	1.00	0.97	0.90
	Moderate wear	0.90	0.96	0.93	
	Severe wear	0.54	0.15	0.23	
	Macro Avg	0.79	0.70	0.71	
PG-MGAF	Slight wear	0.99	1.00	0.99	0.94
	Moderate wear	1.00	0.81	0.90	
	Severe wear	0.86	1.00	0.92	
	Macro Avg	0.95	0.94	0.94	

**Table 8:** Quantitative comparison of six RGB channel assignment permutations in PG-MGAF reconstruction under the LOTO protocol.

Method	R Channel	G Channel	B Channel	Avg Accuracy	Avg Macro-F1	Avg Severe Recall
M1	GASF	GADF	OPM	0.936	0.937	0.954
M2	GASF	OPM	GADF	0.877	0.873	0.832
M3	GADF	GASF	OPM	0.897	0.892	0.932
M4	GADF	OPM	GASF	0.858	0.851	0.808
M5	OPM	GASF	GADF	0.864	0.858	0.793
M6	OPM	GADF	GASF	0.909	0.904	0.973

Note: OPM denotes the cross outer product matrix used in the PG-MGAF framework.

## Data Availability

The data are contained within the article.

## References

[1] Sahu A.K., Mahapatra S.S., Martin A., Schubert A., Leite M., Peças P. (2025). Electrical discharge machining by rapid tools prepared by micro stereo-lithography process with copper metallization. Sci. Rep..

[2] Nguyen H.P., Nguyen D.T., Kim J.M. (2026). Gaussian process regression with physics-guided pseudo-sample augmentation for wear prediction under sparse measurements in milling. Sci. Rep..

[3] Sun X., Yang Z., Xia M., Liu C., Zhou Y., Guo Y. (2025). Tool condition monitoring model based on DAE–SVR. Machines.

[4] Xia Y., Zheng G., Li Y., Liu H. (2025). A Spatial–Temporal Adaptive Graph Convolutional Network with Multi-Sensor Signals for Tool Wear Prediction. Appl. Sci..

[5] Chai A., Fang Z., Lian M., Huang P., Guo C., Yin W., Wang L., He E., Li S. (2025). Hi-MDTCN: Hierarchical Multi-Scale Dilated Temporal Convolutional Network for Tool Condition Monitoring. Sensors.

[6] Xu Y., Li R., Liu X., Liu H., Wang Y., Liu X., Gan Y. (2026). Multi-Scale Dual-Attention Feature Network with Bidirectional Temporal Constraints for Tool Wear Monitoring. Coatings.

[7] Zhou J., Liu X., Liao Q., Wang T., Wang L., Yang P. (2025). Multi-sensor heterogeneous signal fusion transformer for tool wear prediction. Sensors.

[8] Luo G.T., Kuo R.J. (2026). Integrating multi-sensor signal data imaging and convolutional neural network regression model for tool wear monitoring. Int. J. Adv. Manuf. Technol..

[9] Zhao H., Han S., Geng J., Han Y., Jia S., Li K. (2025). MSF-DETR: A small target detection algorithm for sonar images based on spatial-frequency domain collaborative feature fusion. PLoS ONE.

[10] Shi P., Yu Y., Gao H., Hua C. (2022). A novel multi-source sensing data fusion driven method for detecting rolling mill health states under imbalanced and limited datasets. Mech. Syst. Signal Process..

[11] Zhang F., Gao B., Wang Y., Guo L., Zhang W., Xiong X. (2025). MSIMG: A Density-Aware Multi-Channel Image Representation Method for Mass Spec-trometry. Sensors.

[12] Martínez-Arellano G., Terrazas G., Ratchev S. (2019). Tool wear classification using time series imaging and deep learning. Int. J. Adv. Manuf. Technol..

[13] Zhang Y., Qi X., Wang T., He Y. (2023). Tool Wear Condition Monitoring Method Based on Deep Learning with Force Signals. Sensors.

[14] Liu E., Liu C., Du Y., Zhu B., Shi L. (2026). Research on Tool Wear State Recognition Method Based on Multi-Scale Feature Extraction and Deep Residual Network Fusion. Meas. Sci. Rev..

[15] Li S., Li M., Gao Y. (2025). Deep Learning Tool Wear State Identification Method Based on Cutting Force Signal. Sensors.

[16] Yu H., Yang R., Liu H., Du W., Zhang J., Han Z. (2025). Tool wear state prediction based on GAF-MTF-AlexNet. Int. J. Adv. Manuf. Technol..

[17] Zhou Y., Sun W., Ye C., Peng B., Fang X., Lin C., Wang G., Kumar A., Sun W. (2023). Time-frequency representation-enhanced transfer learning for tool condition monitoring during milling of Inconel 718. Eksploat. Niezawodn..

[18] Sun W., Zhou J., Sun B., Zhou Y., Jiang Y. (2022). Markov Transition Field Enhanced Deep Domain Adaptation Network for Milling Tool Condition Monitoring. Micromachines.

[19] Kou R., Lian S., Xie N., Lu B.-E., Liu X.-M. (2022). Image-based tool condition monitoring based on convolution neural network in turning process. Int. J. Adv. Manuf. Technol..

[20] Wang Z., Oates T. (2015). Imaging time-series to improve classification and imputation. arXiv.

[21] Qi S., Ma Y., Liu Z., Jia L., Wu H., Song X., Ning X. (2025). Evaluating the effect of OPM array errors on the multiple dipole localization accuracy of OPM-MEG. Measurement.

[22] Wan X., Chen F., Mo D., Sun Z., Hu K., He Y. (2025). CSA-Kansformer: Cross-scale aggregation and Kansformer network for hyperspectral image classification. Neural Netw..

[23] De Barrena T.F., Ferrando J.L., García A., Badiola X., De Buruaga M.S., Vicente J. (2023). Tool remaining useful life prediction using bidirectional recurrent neural networks (BRNN). Int. J. Adv. Manuf. Technol..

[24] Wu J., Huang Z., Thoma J., Acharya D., Van Gool L. Wasserstein divergence for gans. Proceedings of the European Conference on Computer Vision (ECCV).

[25] Fang X., Song Q., Qin J., Li Z., Ma H., Liu Z. (2025). A dual knowledge embedded hybrid model based on augmented data and improved loss function for tool wear monitoring. Robot. Comput.-Integr. Manuf..

